# Altering gillnet soak duration and timing minimizes bycatch and maintains target catch

**DOI:** 10.1371/journal.pone.0325725

**Published:** 2025-06-25

**Authors:** Sydney M. Collins, Robert J. Blackmore, Jessika Lamarre, Caleb S. Spiegel, William A. Montevecchi

**Affiliations:** 1 Departments of Biology and Psychology, Memorial University of Newfoundland, St. John’s, Newfoundland and Labrador, Canada; 2 Migratory Birds Program, United States Fish and Wildlife Service Northeast Region, Massachusetts, United States of America; Hawaii Pacific University, UNITED STATES OF AMERICA

## Abstract

Seabirds are one of the most at-risk avian groups worldwide, and incidental catch in fishing practices is one of the top threats for seabirds globally. Seabirds that forage on fish through surface feeding, pursuit-diving, or plunge-diving are particularly vulnerable to bycatch. Bycatch mitigation solutions are therefore a vital component of global seabird conservation, but owing to the episodic nature of bycatch and its involvement of match-mismatch contingencies, results from existing efforts involving gear additions (e.g., lights, flags, or buoys) are highly varied and, at times, reduce target catch. Altering the time during which gear remains in the water and modifying fishing practices based on the activity patterns of target fish and seabirds is a promising option for bycatch mitigation. We experimentally tested best practices for the soak timing and duration of shallow-set gillnets used in the Atlantic herring (*Clupea harengus*) bait fishery in Newfoundland and Labrador, Canada. We compared catch, bycatch, and seabird activity among control (ca. 24 h) and short (ca. 12 h) set durations that were left to soak overnight or only during daylight hours. Target catch did not differ between control and short overnight sets but was greatly reduced during short daytime sets. Nearly all bycatch, including all seabird bycatch, occurred during the control sets. Seabirds associated with fishing vessels throughout the day. Since the catch of herring in gillnets occurs at night outside of most coastal seabirds’ foraging period, we recommend that fishers continue to haul their nets early every morning to minimize the time where shallow-set nets are filled with prey during daytime hours, thereby limiting seabird bycatch risk.

## Introduction

Seabirds are among the most at-risk avian taxa worldwide, with 47% of seabird species currently in decline [1]. While this decline is multifaceted, bycatch (the incidental catch of non-target species) in fishing gear is one of the top two threats for seabirds [1,2], whereby diving and surface-feeding seabirds become entangled and drown [3,4]. Groups such as procellariiformes, suliformes, anseriformes, and charadriiformes that forage on fish or offal through surface feeding, pursuit-diving, or plunge-diving methods are particularly vulnerable to bycatch [2,3,5–7]. Over 80 seabird species are at risk of bycatch, with gillnets posing the largest risk [1,8,9]. For example, it is estimated that between 1,580 and 11,500 seabirds are caught annually in the Norwegian coastal gillnet fishery [3]. Globally, gillnet bycatch is thought to cause 400,000 to over a million seabird deaths each year [6,10].

Although other gear types, such as handlines, seines, and pots, produce less bycatch [11], fishers tend to resist gear changes due to cultural, logistical, or economic reasons [12]. Thus, there is a need to develop simple bycatch mitigation strategies to overcome these barriers. Modifying existing gear types and current fishing methods in such a way that could be broadly accepted would have a higher likelihood of being implemented into fishing practices.

Numerous strategies have been employed to reduce seabird bycatch in gillnets, yet no known comprehensive and effective solution has been found [9]. Visual cues have been proposed as a strategy to render gillnets more visible to birds [13,14]. For example, Martin and Crawford [13] designed high-contrast black and white panels that were highly detectable to seabirds foraging underwater. The effects of these panels on seabird bycatch were found to be equivocal [15], and in one case, significantly reduced target fish catch [14], making the panels an untenable gear modification for fishers. Other efforts using light attachments on gillnets provided conflicting information on whether light could reduce seabird bycatch. For example, Mangel et al. [16] reported a significant reduction in the bycatch of Guanay Cormorants (*Leucocarbo bougainvillii*) in gillnets equipped with green LED lights. Yet, follow-up studies using green or white LED lights in the North Atlantic not only found that neither type of lights reduced bycatch in gillnets [15,17], but that white flashing lights actually increased the bycatch of Long-tailed Ducks (*Clangula hyemalis*) [15] and green lights increased the bycatch of Northern Fulmars (*Fulmarus glacialis*), Common Murres (*Uria aalge*), and Northern Gannets (*Morus bassanus*) [18].

Modifying fishing practices according to activity patterns of seabirds and target fish is another option for bycatch mitigation [19]. Some studies have shown that the time of day during which nets are set can mitigate bycatch, since seabirds tend to be less active at night than during the day [20–22]. Traditionally, gillnet fishing practices vary across fisheries, and tend to involve setting gillnets in the morning or afternoon and hauling them 24–48 h later to maximize catches [23]. Weekly quotas come with high fuel costs, so at times, fishers use time-minimizing strategies including setting nets for shorter intervals [for trends, see [24]] and hauling them during a single trip before returning to port, thereby saving a trip, time, resources, and fuel. Shorter soak durations yield higher fish quality, as there is less time for the catch to become moribund while entangled [25]. Shortened soak durations may also reduce the overall risk of bycatch [19,21,26], including for threatened and endangered species (e.g., Atlantic salmon *Salmo salar*, Atlantic cod *Gadus morhua*, porbeagle *Lamna nasus*; 14).

Seabird bycatch in Newfoundland and Labrador, Canada is a known yet understudied conservation concern [27]. Newfoundland and Labrador hosts globally significant populations of Northern Gannets, Common Murres, Atlantic Puffins (*Fratercula arctica*), Black-legged Kittiwakes (*Rissa tridactyla*), and *Larus* gull species [28–31], all of which are vulnerable to gillnet entanglement [3,4,27,32]. Non-breeding shearwaters (*Ardenna* sp.) are also vulnerable to bycatch in gillnets due to their surface-feeding and shallow diving foraging strategies [4], though their occurrence is sporadic prior to July in Newfoundland and Labrador waters [33–36]. Despite seabird abundance and spatiotemporal overlap with major fisheries which drive the economy of Newfoundland and Labrador, no monitoring regulations or efforts have been implemented by regulatory bodies to mitigate or monitor the incidences of seabird bycatch [37].

Among the least monitored of Newfoundland and Labrador’s fisheries is the herring bait fishery. Spanning April until early July [38], fishers use shallow-set gillnets to catch Atlantic herring (*Clupea harengus*) exclusively for use as bait in lucrative shellfish fisheries (American lobster *Homarus americanus* and snow crab *Chionoecetes opilio*) [39]. Bait fishers are not assigned a quota of herring and are not compelled to report information about their fishing operations (e.g., amount of gear deployed, location of deployment, soak duration), catch of herring, or incidences of seabird bycatch [38]. All existing documentation regarding bycatch in the herring bait fishery stems from volunteer-based fisher surveys undertaken by the federal fishing agency [39–41] and one published study [14]. The study documented incidences of seabird and other bycatch. The fisher federal surveys relied on self-reported data, with seabird bycatch widely reported. Seabird bycatch was often not specified to the species level in the surveys, and the magnitude of the problem remains poorly understood. However, the risk of bycatch appears ubiquitous in this fishery with Northern Gannets apparently most at risk [14,39–41].

To better understand and mitigate the risk of seabird bycatch in the herring bait fishery, we studied the target catch, bycatch, and association of seabirds with shallow-set gillnets deployed in the inshore waters of Newfoundland which target Atlantic herring for bait. In collaboration with shellfish harvesters who fish herring for bait, we manipulated the duration and timing of sets and hauls for shallow-set herring gillnets as a potential mitigation technique to reduce seabird bycatch (based on methods from [42]). Whereas herring bait fishers typically set and haul their shallow-set herring gillnets every 24 h [Tony Doyle, pers. comm.; 14], most catch is thought to occur overnight when diurnally-migrating herring rise to the surface to feed [43]. As such, we predicted that shortening the soak duration of gillnets (12 h instead of 24 h) and restricting deployment to nighttime hours (18:00–06:00) might serve as an effective mitigation technique to reduce bycatch while maintaining target catch when compared to control sets left in the water for approximately 24 h between hauling events.

## Materials and methods

### Ethics Approval

All methods were performed under appropriate permits (Canadian Wildlife Service Scientific Permit, number SC2674; Environment and Climate Change Canada Scientific Permit to Capture and Band Migratory Birds, number 10332 K) and were approved by Memorial University of Newfoundland and Labrador’s Animal Care Committee (number 24–01-WM).

### Study sites

**Fig 1:**
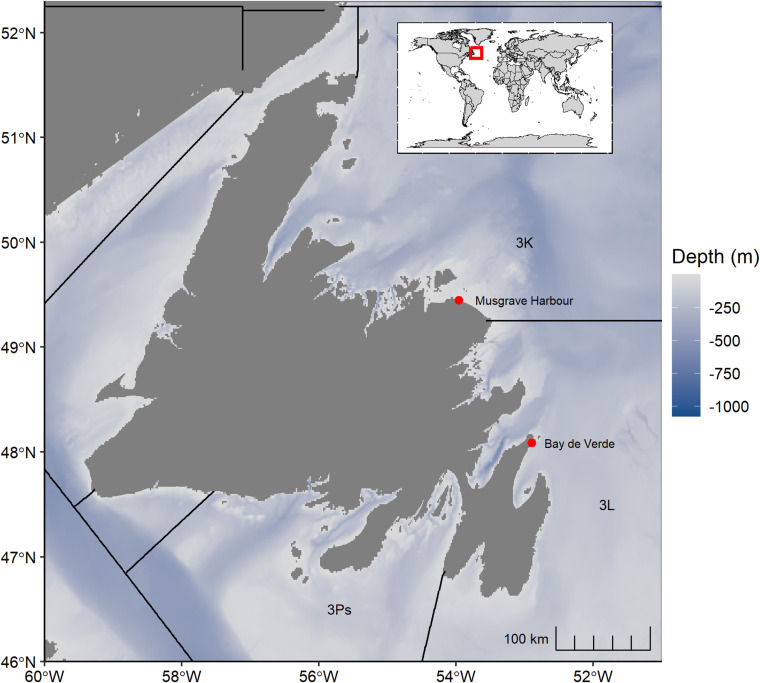
Study locations (red points) and Northwest Atlantic Fisheries Organization (NAFO) regions around the island of Newfoundland, Canada. At Bay de Verde, gillnets were deployed 140 m away from the shoreline where the water depth was 10.5 m on average. At Musgrave Harbour, gillnets were deployed 500 m from the shoreline where water depth reached 13 m. The inset map indicates the global location of the island of Newfoundland. The shapefile for the NAFO regions was obtained from Fisheries and Oceans Canada (https://open.canada.ca/data/en/dataset/59af1c96-fc8f-4fa0-b398-d65e953eadaa). Bathymetric data were obtained from the National Ocean and Atmospheric Administration https://data.noaa.gov/metaview/page?xml=NOAA/NESDIS/NGDC/MGG/DEM//iso/xml/etopo_2022.xml&view=getDataView&header=none). The world map was plotted using the maps package in R (https://cran.r-project.org/web/packages/maps/index.html).

Fieldwork was conducted in Musgrave Harbour and Bay de Verde, Newfoundland and Labrador, Canada (Fig 1), from May 16–27, 2024. During the study, daylight hours in these locations spanned approximately 05:00–20:30. These two locations are in different Northwest Atlantic Fisheries Organization (NAFO) fishing management divisions [44]; 3K and 3L, respectively (Fig 1). The herring bait fishery in each region typically operates from early April to early July [39]. In both regions, the fishery overlaps with the incubation period of Northern Gannets and gulls (*L. smithsonianus, L. marinus, R. tridactyla*) [45–47]. The fishery occurs during the pre-breeding and early incubation period of auks including Atlantic Puffins, Common Murres, and Black Guillemots (*Cepphus grylle*) [28,30,31]. Shearwaters (*A. gravis, A. grisea, Puffinus puffinus*) and Common Eiders (*Somateria mollissima*), may also be present throughout the study areas at low densities during the herring bait fishery season [35,36,48].

### Field methods

**Fig 2:**
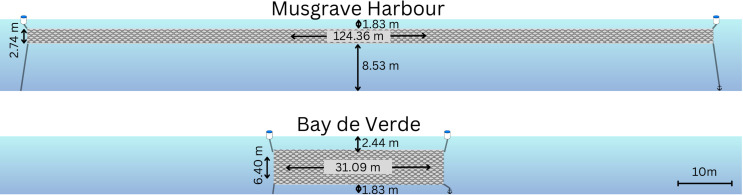
Schematic of the shallow-set herring gillnets deployed at each study location. The nets are illustrated to scale to facilitate visual comparison. In Bay de Verde (total net area of two nets = 397.96 m^2^), each of the two nets deployed were shorter in length but greater in height than those deployed at Musgrave Harbour (total net area of 2 nets = 682.28m^2^). The nets used in each location were the fishers’ own gear used during typical fishing practices at each site. Note that dive depths of alcids and gannets exceed the set depth of all nets in this study.

We worked with crews of commercial shellfish harvesters in open 25’ (7.6 m) fishing boats. Shallow-set herring gillnets (Musgrave Harbour: nominal stretched mesh length = 5.1 cm, net size = 341 m^2^; Bay de Verde: nominal stretched mesh length = 6.7 cm, net size = 199 m^2^; Fig 2) were deployed as part of normal fishing operations in inshore waters. Following federal regulations [49], the nets were deployed parallel to the coastline at a depth of 1.83 to 2.44 m beneath the surface and held vertically in the water column by anchoring them to the seafloor and fitting the head rope with floats (Fig 2). Diving seabirds regularly penetrate below the set depths of these nets [28,45] and are therefore at risk of entanglement throughout the nets’ deployment. As per Musgrave Harbour fishers’ regular practices, two sets of gillnets were deployed simultaneously at a distance of 200 m from each other. At Bay de Verde, only one gillnet was deployed for the first seven trips before a second one was added by the fishers for the remaining nine trips, placed 350 m from the initial net. Sets of gillnets were assigned to one of three soak treatments, where gillnets were left to soak for a day or more within the control treatment (typical fishing practices) or were hauled after a reduced soak duration which was restricted to daytime hours (reduced day treatment) or nighttime hours (reduced night treatment; Table 1). At both sites, set treatments were typically alternated between control and reduced, but due to weather constraints, treatments occurred opportunistically [see metadata in [50]]. It is unlikely that the phenology of any seabird in our study would have influenced the presence or absence of that species in our study area throughout the short temporal spread of our treatments (11 days). When weather allowed, both reduced sets (day and night) occurred within the same 24 h period. As per the normal fishing practices of Newfoundland and Labrador, nets were simultaneously hauled and redeployed (see S1 File).

**Table 1:** Description of study treatments for soak duration and time deployed on shallow-set gillnets in the inshore waters of Musgrave Harbour (MH) and Bay de Verde (BdV), Newfoundland and Labrador, Canada.

Treatment	Soak duration (mean ± SD)	Soak duration (min-max)	Time of gear deployment (min-max)	Sample size (MH, BdV)
Control (typical fishing practices)	28.1 ± 9.1 h	18.7 - 48.0 h	Morning (06:16–10:59) or afternoon (16:10–18:45) depending on weather	15 (6, 9)
Reduced Day	10.3 ± 1.4 h	7.7 - 11.8 h	06:09 − 07:30	11 (6, 5)
Reduced Night	13.7 ± 2.1 h	11.8 - 16.5 h	13:30 − 18:45	8 (5, 3)

During each set and haul, we recorded the date, time of day, weather, ocean conditions, fishing location (latitude, longitude), total catch (number of target fish), total bycatch (number of each non-target species, including non-seabirds), and seabird abundance within close enough proximity of the vessel that they could be identified to species (ca. 200 m in clear conditions, or less in foggy conditions, as in [14]). Seabirds were counted during the hauling period of the simultaneous hauling and re-setting of nets (see S1 File). Upon docking, we measured the fork length (cm) and mass (g) of up to 25 randomly selected Atlantic herring to examine possible differences in catch size among treatments and to estimate total catch mass. On two occasions, we were unable to measure a subsample of herring, so total catch mass was estimated from the average mass calculated at each site. Bycatch was identified to species onboard the vessels, with photographs taken of uncertain species such as sculpins, to be identified at a later time with reference to identification guides [51,52].

### Statistical methods

All analyses were conducted in R version 4.4.2 [53]. Models were constructed with glmmTMB [54]. All model assumptions were verified using the package DHARMa [55] to ensure that the residuals met the assumptions of normality and homogeneity of variance.

### Variation in target catch

The dimensions and number of gillnets set per trip differed between study sites, so we calculated target catch weight (kg) per area of net deployed (m^2^) for each set as a measure of catch per unit effort (hereafter *Scaled Target Catch*). First, we tested whether the scaled target catch (kg/m^2^) differed among the three *Soak Treatments* (categorical: control, reduced day, reduced night) using a generalized linear model with a Tweedie distribution and log link while controlling for the study sites (*Site*; categorical: Musgrave Harbour, Bay de Verde), and including date as a random intercept (*Day of Year*). Benjamini-Hochberg method post-hoc tests [56] fit with the emmeans package [57] investigated the difference in scaled target catch among the three soak treatments.

### Variation in bycatch

Seabird bycatch is highly episodic [9] and our sample size was limited by the brevity of the herring fishing season and the availability of fishers willing to experimentally manipulate their fishing practices and report bycatch. The statistical power to assess variation in bycatch of our study is therefore highly limited, and so we present descriptive statistics regarding the incidence of bycatch. We created a boxplot of the number of incidentally caught individuals of any species (seabirds and non-seabirds) for each soak treatment. We also plotted the number of incidentally caught individuals against the soak duration in hours to visualize associations between soak duration and bycatch risk.

### Variation in seabird abundance

Because herring gillnets are set near the water surface, diving seabirds are at risk of entanglement throughout the nets’ deployment, including during setting and hauling. Surface-feeding seabirds are vulnerable to bycatch during hauling when the nets are filled with fish and pulled to the surface (see S1 and S2 Files). Birds were only counted at the time of hauling, rather than systematically throughout the day. We examined the association between time of hauling and the number of birds present near the vessel using a scatterplot.

## Results

### Variation in target catch

**Fig 3:**
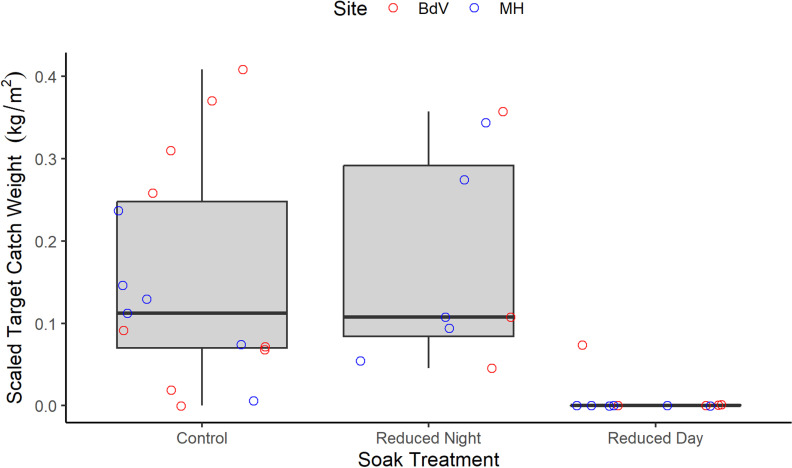
Variation in scaled target catch weight (kg of herring caught per m^2^ of a shallow-set gillnet) among the different soak time and duration treatments. Raw data are represented by the points, coloured by site (Bay de Verde (BdV) and Musgrave Harbour (MH)) and grouped by soak treatment. The control treatment refers to gillnets set for >18 h before hauling (in the water during both daytime and nighttime hours; n = 15), whereas the reduced treatments refer to gillnets set for <16 h, either during daylight hours (reduced day; n = 11) or during nighttime (reduced night, n = 8).

Scaled target catch significantly differed among the study treatments, where catch was significantly lower during the reduced day sets, but did not differ among the control and reduced night treatments (mean ± SD: control = 0.15 ± 0.13 kg/m^2^, reduced night = 0.17 ± 0.13 kg/m^2^, reduced day = 0.01 ± 0.02 kg/m^2^; full model results for Soak Treatment: χ^2^ = 25.94, df = 2, p < 0.001; BH corrected post hoc tests: control – reduced night: z_ratio_ = −0.54, p = 0.593; control – reduced day: z_ratio_ = 4.70, p < 0.001; reduced night – reduced day: z_ratio_ = 4.97, p < 0.001; Fig 3, S1 Table). Scaled target catch did not differ between sites (χ^2^ = 1.32, df = 1, p = 0.250).

### Variation in bycatch

**Fig 4:**
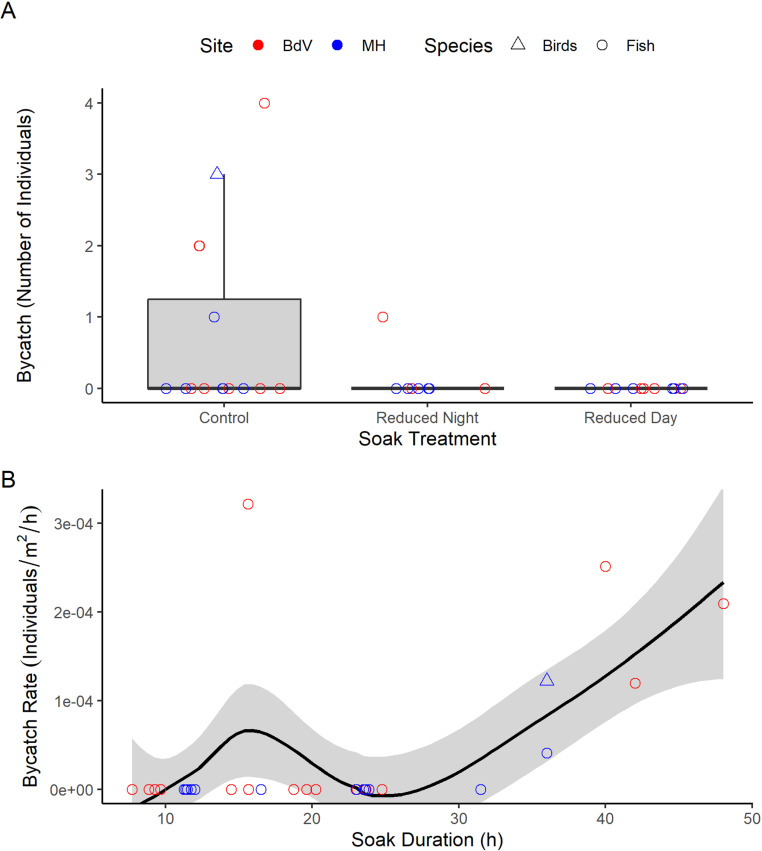
The number of non-target animals caught (“Bycatch”) in shallow-set Atlantic herring (*Clupea harengus*) gillnets was higher with greater soak durations. Raw data are represented by the points, coloured by study sites (Bay de Verde (BdV): N = 17 hauls; Musgrave Harbour (MH): N = 17 hauls). Triangles represent seabird bycatch events, and circles represent the fish bycatch events. **A)** Almost all incidences of bycatch occurred during control treatment sets where soak duration lasted a day or longer, including the gannet bycatch event. **B)** Bycatch rate tended to be higher following longer soak durations. The black line is the locally weighted smoothing (LOESS) line and the grey area is its 95% confidence interval.

Five bycatch events occurred during our study, totalling 10 finfish and three seabirds (Table 2, Fig 4). Finfish were caught during all five bycatch events, whereas all seabirds were caught within a single haul (Table 2, Fig 4a, S1 Fig). Four of the five bycatch events, including the haul where three seabirds were found entangled in a net, occurred during our control treatment. All bycatch events observed in control sets occurred when nets had been left to soak for >35 h (Fig. 4b). Only one instance of bycatch occurred during a reduced soak treatment (reduced night; Table 2, Fig 4a). The main species incidentally caught included Atlantic cod and shorthorn sculpin (*Myoxocephalus scorpius*; S1b Fig), as well as three Northern Gannets which were found side-by-side in the net during the same hauling event (S1c-d Fig). The gannets were caught in a control net that had been reset 36 h prior (18:00 set time, 06:00 haul time) since inclement weather prevented hauling at the 24 h mark; strong winds, rain, and rough swells occurred during the day when the nets would have been left in the water, presumably filled with herring from the previous night.

**Table 2:** Sum of incidental catch in shallow-set herring gillnets deployed under manipulated soak timing and duration treatments.

Bycatch	Control N = 15	Reduced day N = 11	Reduced night N = 9
**Finfish**			
Atlantic cod (*Gadus morhua*)	4	0	1
Shorthorn sculpin (*Myoxocephalus scorpius*)	4	0	0
Yellowtail flounder (*Limanda ferruginea*)	1	0	0
**Seabirds**			
Northern Gannet (*Morus bassanus*)	3^a^	0	0
**Total finfish**	9	0	1
**Total seabirds**	3	0	0
**Grand total**	12	0	1

Bycatch from trips undertaken at both study sites (Bay de Verde and Musgrave Harbour). The control trips refer to normal fishing conditions with nets left to soak for a day or more, while the reduced treatments refer to nets set for ca. 12 h before hauling, either during daytime hours (06:00–18:00 = reduced day) or during nighttime (18:00–06:00 = reduced night).

^a^Caught during a single haul

### Variation in seabird abundance

**Fig 5:**
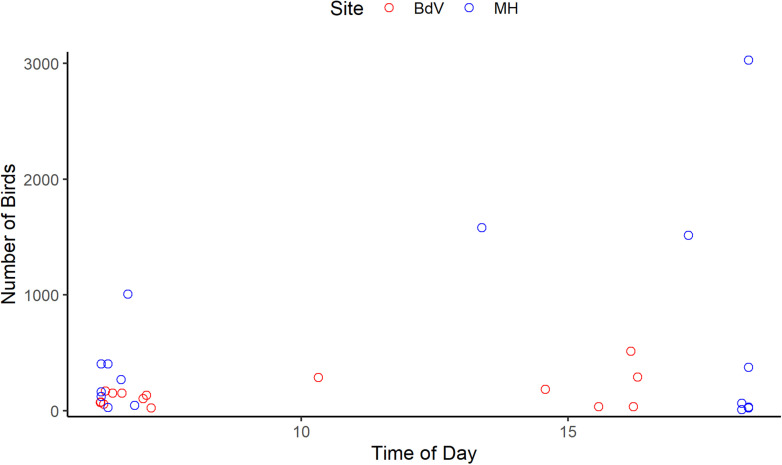
Seabird abundance near the fishing vessel varying with time of day. Raw data are represented by open circles, coloured by site (Bay de Verde (BdV): N = 15; Musgrave Harbour (MH): N = 18).

At the time of hauling the gillnets, there was considerable variation in the number of seabirds and taxa observed in proximity of the fishing vessel, though seabirds were consistently observed throughout daylight hours (05:00–20:30; Fig 5). More seabirds were observed at Musgrave Harbour than at Bay de Verde (Fig 5, S2 Table), which is a result of a large group (1000s) of gannets foraging for spawning herring in Musgrave Harbour between 20–23 May.

## Discussion

Reducing the duration of time that nets filled with fish are left to soak in the water could be an effective way to mitigate bycatch, including seabird bycatch, without affecting target catch. First, we found that herring catch was the same between the control treatment (in which nets were generally deployed for a 24 h period or longer) and the reduced night treatment (when nets were only deployed overnight and hauled in the morning; S1 Table, Fig 3), showing that herring catch occurs at night. Second, though our sample size was small, several bycatch events were observed, occurring almost exclusively during the control treatment, and mainly under soak durations exceeding 30 h (Table 2, Fig 4). No bycatch occurred within the reduced day treatment (Table 2, Fig 4), and the catch of target herring was virtually null, (S1 Table, Fig 3). Thus, bycatch most likely occurred when nets were full (i.e., had catch that was yet to be hauled). Finally, both diving and surface-feeding seabirds were observed in close proximity to the gillnets throughout daylight hours during hauls and resets (S2 Table, Fig 5), also suggesting that seabird bycatch risk is highest when nets are full of catch (here, herring) and are left to soak throughout the day.

Atlantic herring are planktivorous diel migrants which spend much of their time inshore during the spring and fall spawning seasons [38,58,59]. During this time, herring are at depth near the ocean floor during daylight hours, only rising to the surface at night to feed on planktonic organisms throughout the upper portions of the water column [60–62]. Our study overlapped with an active period of herring spawning at both sites. During such spawning events, schools of Atlantic herring travel from pelagic waters during the night and stagger their arrival at coastal sites, where they spend a few sedentary days spawning [63,64]. Herring remain deep in the water column during the day and while at their spawning grounds, however they swim near the surface during the night as they travel to, from, and within pelagic waters to benefit from higher water temperatures and to feed on zooplankton [58,62,65]. The diurnal patterns of vertical space use, where most of the herring’s movement occurs at night and near the surface, explains why nearly all target catch in this study occurred within control and reduced night treatments. Importantly, we found that fishers do not benefit from longer soak durations as the number of herring caught did not differ across the reduced night and control treatments. Our results suggest that target catch is maximized during the first night of deployment. This might be due to the phenomenon of saturation where a large number of fish are caught in a gillnet simultaneously, rendering the net visible to conspecific schools that then avoid the area [66–69].

We had more bycatch from hauls with longer soak durations (Table 2, Fig 4). Four of the five instances of bycatch observed occurred within the control treatment, specifically in gillnets with soak durations exceeding 30 h (see Fig 4b) when the gillnets were deployed around 18:00 for an overnight set, spent a full day and night in the water, and were subsequently hauled the following morning. These extended soak durations occurred due to severe weather conditions (high wind and rough waters) which prohibited safe hauling at the experimentally intended times. Turbulent water conditions could also have compromised the ability of Northern Gannets – the only seabird bycatch in our study – to detect free-swimming prey within the water column, possibly making herring caught in a gillnet a visibly attractive target. One instance of bycatch occurred during our reduced night treatment (one Atlantic cod), and these gillnets retained as much target catch as the control treatment (see Fig 3b). Within our reduced day treatment, target catch was significantly reduced and no bycatch occurred. Although our sample size was small, these results suggest that shallow-set herring gillnets pose the greatest threat for bycatch when the nets contain target catch with most fish accumulating at night and left to soak during daylight hours. Bycatch is episodic by nature and is thought to occur on a match-mismatch basis, meaning that bycatch can only occur when there is spatiotemporal overlap between non-target species and fishing gear. However, target species that are important to both fisheries and as prey species to a wide scope of predators can lead to concentrations of predators and fishing gear around aggregations of the same prey species, heightening the risk of bycatch. Results like the ones found in this study have been observed in other fisheries where bycatch is more likely to occur when fish are present in the fishing gear since a high density of prey constrained to a given area attracts predators [70–74]. At both Musgrave Harbour and Bay de Verde, sunlight permeates the full water column during the day as water depth is less than 13 m at both sites. Therefore, herring caught in the nets would be visible to both underwater and aerial predators during daylight hours, leaving predators vulnerable to bycatch when they attempted to take fish from the nets.

All three fish species incidentally caught during this study (Atlantic cod, shorthorn sculpin, yellowtail flounder *Limanda ferruginea*) are groundfish which forage mainly during the day but are active at night [75–77]. Studies investigating bycatch mitigation for such demersal species have focused mainly on bottom-dwelling gear, including trawls that drag along the sea floor [78] or bottom-set gillnets that rest on the seafloor and extend vertically to 2 m [79]. Here we show that near-surface gillnets can also present a risk of entanglement to groundfish, congruent with the findings and species compositions of previous research [39,40]. This risk likely relates to the distance of the net from the sea floor, as the bycatch of groundfish occurred several times at Bay de Verde, where nets extended down to 1.8 m from the seafloor given the shallow nature of the deployment site (<10.5 m). Cod caught during this study were large adults (see S1b Fig), likely preying on herring near the shallow coastline [62]. Cod bycatch is concerning as Northwestern stocks of Atlantic cod remain under duress following a large-scale collapse during the early 1990s [80]. Monitoring efforts of population health have not investigated how problematic bycatch of cod in pelagic fisheries might be for the species, and fishers are unlikely to report occurrences of cod bycatch to regulatory bodies for fear of jeopardizing the commercial cod fishery [4,38,39]. Surprisingly, no regulation exists in Newfoundland and Labrador to oversee the depth at which shallow-set gillnets might extend [38,39], which is left at the discretion of the fishers. As a result, the bycatch risk of herring gillnets is difficult to predict as it may vary both by region and fisher. Further, there are many Canadian fisheries for which there is no mandate to collect bycatch data (collection of seabird bycatch data is not required in any Canadian fishery [37]). Additionally, there is no enforcement of mandatory reporting regulations (e.g., reporting of species such as cod, sharks, and other listed species is often lacking as is true for the herring bait fishery [38,39]).

The bycatch of breeding adult Northern Gannets documented in our study (see S1 Fig) is consistent with the limited literature on the Newfoundland herring bait fishery [14,39–41], and highlights one of several substantial threats the species encounters throughout its range. Northern Gannet population sizes and growth rates in North America have decreased due to factors that impact survival rate (e.g., avian influenza [81–83], oil spills [84–86]) and reproductive success (e.g., nutritional stress caused by climate change and overfishing [29,87,88], invasive predators [89,90], avian influenza [82,91]). Gannets tend to increase their rates of plunge-diving in turbid waters [92], relying on their vision to detect fish once in the water column. Accordingly, the bycatch event that resulted in gannet mortality during this study occurred during a control set that lasted 36 h due to stormy weather; all conditions were met to create a high entanglement risk event, with nets filled with herring soaking during the daytime in rough, turbid waters. Such adverse weather conditions are common in Newfoundland and Labrador and do not typically deter fishers from fishing but may cause fishers to leave their nets to soak for extended periods since the quality of their catch does not impair its usage as bait in shellfish traps. The temporal diet patterns of Northern Gannets may have put them at an elevated risk for bycatch during our study, as breeding gannets in the northwestern Atlantic rely on herring as a dominant food source for themselves and their young chicks in their early breeding season (May to early August) [93–95].

We found that several bird species associated with the gillnets throughout daylight hours (S2 Table, Fig 5). All gannets, alcids, gulls, cormorants, and seaducks observed during this study are diurnal, and the start and end of their active foraging period (during crepuscular hours) coincides with herring in the upper range of the water column at dawn and at dusk [22,60–62]. These birds forage throughout the day and tend to travel further during the afternoon [96–98]. While not a direct predictor of bycatch, evaluating the number of birds in the vicinity of fishing gear when hauling provides an indication of potential bycatch based on species abundance and behaviour towards the gear [14,99]. For example, the potential for gannet bycatch during our study was high in Musgrave Harbour due to their sheer abundance (S2 Table) in proximity to a significantly larger gannet colony (Funk Island) than the smaller colony (Baccalieu Island) near Bay de Verde [100]. Bycatch risk is linked to herring spawning events near the study site because gannets plunge-dive at depths exceeding the nets’ set-depth [45], putting them at risk during the gillnets’ entire deployment. Bycatch risk of surface-feeders (e.g., gulls) was likely concentrated during hauling when nets were full of herring, since the gillnet catch is only accessible to them when pulled to the surface. Indeed, gulls were sighted in the vicinity of the gillnets during every trip at both sites and were observed diving on the nets and successfully extracting herring from them during hauling (see S2 File); in previous studies, this behaviour has been reported to lead to surface-feeder entanglement in gillnets [3].

The bycatch of gannets and other seabird species is often episodic [9] such that long-term systematic monitoring of bycatch incidents is required to obtain a comprehensive understanding of how problematic a given fishery might be for a given species. Such data do not exist for the herring bait fishery. Alarmingly, incidences of bycatch are thought to have increased in recent years following a sharp increase in herring bait gillnet deployment throughout eastern Canada [101] related to increased lobster fishing effort. Lobster landings have grown exponentially in Newfoundland from 3,340 MT in 2018 [102] to 10,115 MT in 2024 [103]. Currently, there is no quota established for the lobster fishery in Newfoundland [102,103] and as such, we do not expect the demand for bait to decrease unless lobster catch rates start decreasing.

Based on our findings that: 1) herring catch primarily occurred at night, 2) bycatch of both non-target fish and of seabirds was associated with soak times >24 hours, and 3) seabirds were abundant near vessels throughout daylight hours, we recommend that fishers minimize the soak duration of their shallow-set gillnets by setting at night and hauling the following morning at first light. Where this is not practical due to additional boat trips and associated operational costs and efforts, we recommend hauling and resetting shallow-set herring gillnets in the early morning at least every day. Given our small sample size and the episodic nature of seabird bycatch, we also recommend widespread monitoring of herring bait fishery practices and bycatch to better understand the magnitude of fishery impacts on incidentally caught species.

We stress that our findings are gear- and species-specific, in terms of the target and incidentally caught species. For example, Savina et al. [42] reported contrasting results to ours, when deploying bottom-set gillnets targeting European plaice (*Pleuronectes platessa*) over reduced night sets (12 h overnight) halved their overall catch relative to 24 h sets, whereas daytime 12 h sets yielded larger target species and reduced crab bycatch. This led the authors to recommend 12 h soak times during the day as the best practice specific to that fishery [42]. This result highlights the importance of trialing soak time manipulations in various fisheries globally, as best practices for soak duration and time likely vary among target species, common bycatch species, gear types, location, and local standards of practice. Manipulating the timing and duration of gear soaks is a straightforward treatment that can be easily applied to the use of any passive gear type, and can be used as an effective way to ensure the quality of target catch and restrict interactions of the gear with non-target species, reducing the possibility of bycatch and lost gear [104].

## Conclusions

Our results suggest that hauling shallow-set herring gillnets in the early morning may reduce bycatch without compromising target catch, facilitating maximal nighttime herring catch while reducing the duration that nets full of herring are left to soak during daylight hours when predators are actively foraging. These results allow us to suggest best-practices for the herring bait fishery, where the sole regulations pertaining to mitigating bycatch mandate fishers to set their gillnets at least 1.83 m below the surface and parallel to the shore to reduce the risk of Atlantic salmon bycatch [38,39]. We acknowledge, however, that our “reduced overnight” soak treatment, where nets are set in the evening and hauled in the morning, may not always be a practical approach that fishers could easily or willingly implement under normal circumstances due to the increased effort and associated costs. As such, we purport that maintaining a maximum 24 h soak time and ensuring that the nets remain empty during daylight hours by hauling in early morning would be the most realistic yet effective practice to implement. This method is not different from the standard practice of many fishers in Newfoundland and Labrador, meaning it is a feasible and agreeable recommendation to implement in an active fishery.

## Supporting information

S1 Fig Deployment of shallow-set gillnets targeting herring during the spring fishery in the Northwestern Atlantic. A) Gillnets filled with herring after an overnight soak and attracting several seabirds (Larus spp. shown here) during hauling. B) Atlantic cod incidentally caught in a gillnet otherwise filled with herring. C) Three Northern Gannets (two shown here) were incidentally caught in a gillnet during a single bycatch event following an extended soak duration caused by stormy weather. D) The three Northern Gannets caught were all sexually mature adults.
(TIF)

S1 Table Mean catch number, catch mass, individual mass, and individual fork length of Atlantic herring quantified by treatment for each site. (DOCX)

S2 Table Number of individuals from each seabird species associating within proximity of the shallow-set gillnets during hauling summed over all trips undertaken at each study site (Bay de Verde and Musgrave Harbour). Morning hauls occurred between 05:45 and 10:30 whereas afternoon hauls occurred between 13:30 and 18:30. The seabird species are grouped by feeding strategy where divers refer to birds that can pursue fish several meters underwater whereas surface-feeders refers to the species that can only access organisms within a few centimeters of the surface.
(DOCX)

S1 File Video of the process where shallow-set gillnets are hauled and redeployed simultaneously. (MP4)

S2 File Video of gulls actively foraging on Atlantic herring caught in a shallow-set gillnet. (MP4)
