# Supplementary figures and images for: Altering gillnet soak duration and timing minimizes bycatch and maintains target catch
Source: PLoS One. 2025 Jun 25;20(6):e0325725. doi: 10.1371/journal.pone.0325725 (PMC12193576; doi:10.1371/journal.pone.0325725)

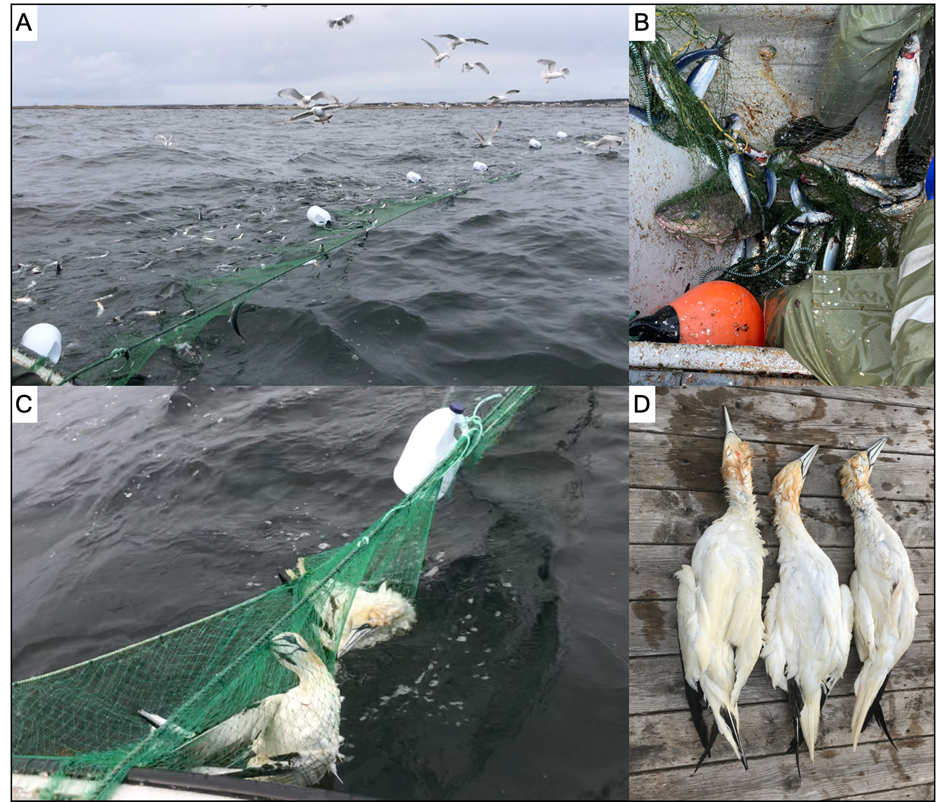

Supplement: S1 Fig — A) Gillnets filled with herring after an overnight soak and attracting several seabirds (Larus spp. shown here) during hauling. B) Atlantic cod incidentally caught in a gillnet otherwise filled with herring. C) Three Northern Gannets (two shown here) were incidentally caught in a gillnet during a single bycatch event following an extended soak duration caused by stormy weather. D) The three Northern Gannets caught were all sexually mature adults. (TIF) [file pone.0325725.s001.tif]
