# Supplementary material for: Altering gillnet soak duration and timing minimizes bycatch and maintains target catch
Source: PLoS One. 2025 Jun 25;20(6):e0325725. doi: 10.1371/journal.pone.0325725 (PMC12193576; doi:10.1371/journal.pone.0325725)
Supplement: S1 Table — (DOCX) [file pone.0325725.s002.docx]

**S1 Table. Mean catch number, catch mass, individual mass, and individual fork length of Atlantic herring quantified by treatment for each study site.**

| **Site** | **Treatment** | **Mean**  **Total Herring Catch (n fish)** | **Mean**  **Total Herring Catch Mass (kg)** | **Mean Individual Herring Mass (g)** | **Mean Individual Herring Fork Length (cm)** |
| --- | --- | --- | --- | --- | --- |
| Bay de Verde | Control | 236.0 | 51.2 | 277.2 | 30.6 |
|  | Reduced Day | 21.6 | 6.0 | 268.0 | 30.2 |
|  | Reduced Night | 248.0 | 60.6 | 263.0 | 29.9 |
| Musgrave Harbour | Control | 356.0 | 62.5 | 175.4 | 26.8 |
|  | Reduced Day | 0.2 | 0.03 | 170.0 | 26.0 |
|  | Reduced Night | 645.0 | 113.0 | 177.8 | 26.8 |
