# Supplementary material for: Altering gillnet soak duration and timing minimizes bycatch and maintains target catch
Source: PLoS One. 2025 Jun 25;20(6):e0325725. doi: 10.1371/journal.pone.0325725 (PMC12193576; doi:10.1371/journal.pone.0325725)
Supplement: S2 Table — Morning hauls occurred between 05:45 and 10:30 whereas afternoon hauls occurred between 13:30 and 18:30. The seabird species are grouped by feeding strategy where divers refer to birds that can pursue fish several meters underwater whereas surface-feeders refers to the species that can only access organisms within a few centimeters of the surface. (DOCX) [file pone.0325725.s003.docx]

**S2 Table. Number of individuals from each seabird species associating within proximity of the shallow-set gillnets during hauling summed over all trips undertaken at each study site (Bay de Verde and Musgrave Harbour).** Morning hauls occurred between 05:45 and 10:30 whereas afternoon hauls occurred between 13:30 and 18:30. The seabird species are grouped by feeding strategy where divers refer to birds that can pursue fish several meters underwater whereas surface-feeders refers to the species that can only access organisms within a few centimeters of the surface.

| **Species sighted** | **Bay de Verde** | | **Musgrave Harbour** | |
| --- | --- | --- | --- | --- |
|  | **Morning**  **N=10** | **Afternoon**  **N=5^a^** | **Morning**  **N=8** | **Afternoon**  **N=8** |
| **Divers** |  |  |  |  |
| Northern Gannet  *Morus bassanus* | 97 | 8 | 2006 | 6385 |
| Cormorant spp. *Phalacrocorax spp.* | 12 | 14 | 52 | 34 |
| Black Guillemot  *Cepphus grylle* | 23 | 15 | 0 | 4 |
| Atlantic Puffin  *Fratercula arctica* | 29 | 0 | 2 | 1 |
| Common Murre  *Uria aalge* | 18 | 0 | 0 | 8 |
| Razorbill  *Alca torda* | 1 | 0 | 0 | 0 |
| Common Eider  *Somateria mollissima* | 121 | 0 | 0 | 0 |
| **Surface-feeders** |  |  |  |  |
| Black-legged Kittiwake *Rissa tridactyla* | 133 | 324 | 0 | 0 |
| Herring Gull  *Larus argentatus* | 753 | 677 | 374 | 193 |
| Great Black-backed Gull *Larus marinus* | 42 | 24 | 43 | 6 |
| Ring-billed Gull  *Larus delawarensis* | 2 | 3 | 9 | 9 |
| Lesser Black-backed gull *Larus fuscus* | 1 | 2 | 0 | 0 |
| Iceland Gull  *Larus glaucoides* | 8 | 0 | 0 | 0 |
| Common Tern  *Sterna hirundo* | 1 | 0 | 0 | 0 |
| **Total divers** | 301 | 37 | 2060 | 6432 |
| **Total surface-feeders** | 940 | 1030 | 426 | 208 |
| **Grand total** | 1241 | 1067 | 2486 | 6640 |
| Seabird sightings from trips undertaken at both study sites (Bay de Verde and Musgrave Harbour)  ^a^Seabird observation data could not be recorded during the first and last trip undertaken at Bay de Verde | | | | |
